# Geographical distribution of zooplankton biodiversity in highly polluted running water ecosystems: Validation of fine‐scale species sorting hypothesis

**DOI:** 10.1002/ece3.4037

**Published:** 2018-04-17

**Authors:** Yuzhan Yang, Ping Ni, Yangchun Gao, Wei Xiong, Yan Zhao, Aibin Zhan

**Affiliations:** ^1^ Research Center for Eco‐Environmental Sciences Chinese Academy of Sciences Beijing China; ^2^ University of Chinese Academy of Sciences Chinese Academy of Sciences Beijing China

**Keywords:** high‐throughput sequencing, metazoan zooplankton, nutrient threshold, river ecosystem, species sorting

## Abstract

Dispersal, rather than species sorting, is widely recognized as the dominant driver for determining meta‐community structure at fine geographical scales in running water ecosystems. However, this view has been challenged by a recently proposed “fine‐scale species sorting hypothesis,” where community structure can be largely determined by an environmental gradient formed by local pollution at fine scales. Here, we tested this hypothesis by studying community composition and geographical distribution of metazoan zooplankton in a heavily polluted river—the North Canal River in the Haihe River Basin, China. Analysis of similarity (ANOSIM) showed that the community composition of metazoan zooplankton differed significantly (*p *=* *.001) along the environmental gradient. Ammonium (NH_4_‐N) was the leading factor responsible for changes in zooplankton community structure and geographical distribution, followed by total dissolved solid (TDS), Na, dissolved oxygen (DO) and temperature (T). Variation partitioning revealed a larger contribution of environmental variables (21.6%) than spatial variables (1.1%) to the total explained variation of zooplankton communities. Our results support that species sorting, rather than dispersal, played a key role in structuring communities. Threshold Indicator Taxa ANalysis (TITAN) also revealed significant change points at both taxon and community levels along the gradient of NH_4_‐N, providing further support for the influence of environmental variables on zooplankton communities. Collectively, we validate the fine‐scale species sorting hypothesis when an environmental gradient exists in running water ecosystems at fine geographical scales. However, future studies on interactions between pollutants and zooplankton communities are still needed to better understand mechanisms responsible for the meta‐community dynamics.

## 1 INTRODUCTION

Meta‐community, a set of local communities that are connected by dispersal of multiple interacting species, is an innovative framework to decipher ecological processes and mechanisms underlying species geographical distribution, abundance, and interactions (Castillo‐Escrivà, Aguilar‐Alberola, & Mesquita‐Joanes, 2017; Leibold et al., 2004; Logue, Mouquet, Peter, & Hillebrand, 2011). In aquatic ecosystems, particularly running water ecosystems such as rivers, species sorting and dispersal are two competitive determinants for meta‐community dynamics (Heino, Melo, & Bini, 2015; Lindström & Langenheder, 2012; Logue et al., 2011). The prerequisite of species sorting is the presence of a significant environmental gradient—where habitats are environmentally heterogeneous along geographical scales (Leibold et al., 2004). Local communities are filtered by environmental factors and species occur at environmentally suitable habitats, provided that dispersal is sufficient for species to track environmental variation along an environmental gradient (Heino, Melo, Bini, et al., 2015). However, dispersal, either from active movement of species or passive advection by water currents, acts as a competing process that can homogenize community structure at adjacent localities (Leibold et al., 2004). In aquatic ecosystems, both species sorting and dispersal are interdependent and interact at different geographical scales to shape the composition and distribution of local communities (Logue et al., 2011; Xiong et al., 2017).

However, it is a great challenge to disentangle the relative importance of these two processes on determining local community structure in running water ecosystems (Downes, 2010; Göthe, Angeler, & Sandin, 2013; Xiong et al., 2017). Such a challenge is attributed to several aspects. First, the relative importance of these two processes is largely scale‐ and gradient‐ dependent (Heino, Melo, Siqueira, et al., 2015; Soininen, Korhonen, Karhu, & Vetterli, 2011; Xiong et al., 2017). At large geographical scales such as across basins, species sorting tends to be more important, as environmental gradients often exist at relatively large geographical scales (Heino, Melo, Bini, et al., 2015; Heino, Melo, Siqueira, et al., 2015). At fine scales such as within a stream, dispersal may counteract the influence of environmental heterogeneity, thus contributing more than species sorting to community structure (Moritz et al., 2013; Xiong et al., 2017; Peng, Xiong, & Zhan, 2018). Second, dispersal can be influenced by the capacity and mode of different species in different, even the same, communities (Heino, Melo, Siqueira, et al., 2015), making it more difficult to interpret the influence of dispersal. Species with a high level of active dispersal can move larger distances than animals with the passive dispersal mode (Tesson & Edelaar, 2013). Even for the same species, the effective dispersal distance may vary at different life and/or developmental stages (Benard & McCauley, 2008; Fisher, Bellwood, & Job, 2000). Finally, different types of ecosystems often lead to varied conclusions. For example, species sorting often prevails in stream networks and ponds, while the importance of dispersal increases in coastal and offshore marine systems (Heino, Melo, Siqueira, et al., 2015). Most importantly, all these factors were interconnected in natural ecosystems and coupled by other factors such as climate changes as well as biological interactions among a large number of organisms in communities (Bertani, Ferrari, & Rossetti, 2012; Henriques‐Silva, Pinel‐Alloul, & Peres‐Neto, 2016). Consequently, all of these issues complicate the study of examining the relative importance of species sorting versus dispersal on determining structure of meta‐communities.

With the nature of dendritic structure, directional flow, and a high level of biodiversity, the lotic river ecosystem provides an excellent model to study how these two processes mentioned above interact to determine community structure (Altermatt, 2013; Brown et al., 2011; Xiong, Yang, & Zhan, 2018). Previous studies have shown that at the eco‐region or basin level, species sorting was more important than dispersal in structuring composition and distribution of biodiversity in meta‐communities (Landeiro, Bini, Melo, Pes, & Magnusson, 2012; Xiong et al., 2016). While within a single stream or river, as the environmental heterogeneity largely decreases with the decreased geographical scales, dispersal can largely erase the influence of species sorting and is dominant in structuring meta‐community assemblages (Heino, Melo, Siqueira, et al., 2015). However, an exception was observed in the study of a highly polluted river in the Haihe River Basin, the Chaobai River where species sorting out competed the influence of dispersal to largely determine the zooplankton community assemblages at a fine geographical scale (~200 km; Xiong et al., 2017). Consequently, the “fine‐scale species sorting hypothesis” was proposed in highly polluted river ecosystems—where community structure is largely determined by an environmental gradient formed by local pollution at fine geographical scales (Xiong et al., 2017). This hypothesis is fundamental for answering diverse ecological questions (e.g., ecological effects of pollution on community structure), particularly under the circumstance that environmental pollution has become a serious problem in river ecosystems globally (Dudgeon et al., 2006; Vörösmarty et al., 2010). Although the results from Xiong et al.'s study have renovated the view on these two competing processes at fine geographical scales, the fine‐scale species sorting hypothesis has not been widely tested.

In this study, we used the North Canal River (NCR) to test the “fine‐scale species sorting hypothesis”. NCR originates from the Shahe Reservoir in Beijing, and this 260‐km‐long river has been highly polluted and tremendously altered by an extremely high level of human activities (Heeb et al., 2012). Pollutants derived from land use and pollution sources largely vary in different regions, forming a potential environmental gradient along this river. The major pollutants in the upstream (also known as Wenyu River) mainly come from wastewater treatment plants in Beijing (i.e., point pollution). Of the total upstream discharge, 93% is treated wastewater due to the consumption of 14 million people (70% of Beijing's total population), and 4% is raw sewage (Heeb et al., 2012). The upstream is also the source of pharmaceuticals, and the majority of household chemicals were detected in the middle stream and downstream (Heeb et al., 2012). The middle stream between Beijing and Tianjin is surrounded by a large area of agricultural lands. Nonpoint sources of pollution, such as fertilizers and pesticides derived from farming lands, are the leading pollutants in this section (Shan, Jian, Tang, & Zhang, 2012). The accumulation of pollutants also contributes to the eutrophication in this area. The downstream is composed of two segments: the upper segment runs through Tianjin and receives treated and untreated wastewater, while the lower segment flows into the Bohai Sea, leading to a relatively high level of salinity (Gong & Mei, 2017). In addition, the flow of water current is slow in NCR and does not change very much along the river based on our previous surveys. Consequently, the potential environmental gradient of NCR represents a promising case to test the fine‐scale species sorting hypothesis.

Here, we used metazoan zooplankton collected from NCR to test the fine‐scale species sorting hypothesis. Zooplankton are crucial components of aquatic food webs by producing and structuring the matter, energy, and information fluxes in river ecosystems (Landeiro et al., 2012; Pulliam, 1988). Zooplankton are free‐living organisms to flow with water currents, making them good subjects to test the dispersal hypothesis (Battuello et al., 2016). Here, we analyzed the environmental gradient along NCR, characterized community structure of metazoan zooplankton using high‐throughput sequencing, explored the relationship between environmental variables and community structure, and finally determined the relative importance of the two competing forces (i.e., species sorting vs. dispersal) to geographical distribution of metazoan zooplankton communities.

## 2 METHODS

### 2.1 Study area and sample collection

**Figure 1:**
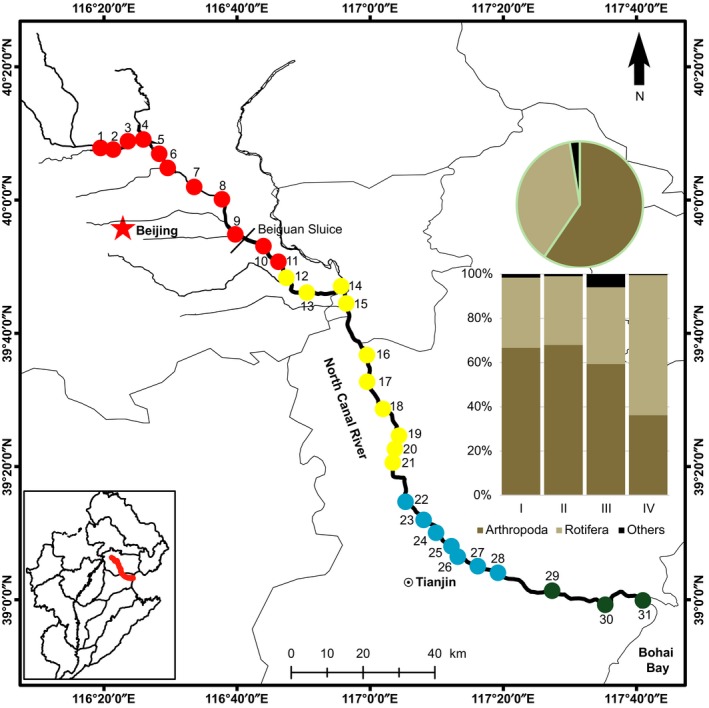
Sampling sites along the North Canal River (NCR). We chose a total of 31 sites along NCR, including eleven in the Section I (red dots), ten in the Section II (yellow dots), seven in the Section III (blue dots), and three in the Section IV (green dots). The pie chart showed the phylum level composition of metazoan zooplankton at all sites, and the histogram showed the phylum level composition in the four sections. The region in the left corner (bottom) is the Haihe River Basin where the NCR is located in

Samples were collected in July 2016 from downstream to upstream of NCR (Fig. 1). NCR was divided into four sections based on significantly contrasting environmental variables (see details in the Results section). Water samples were collected from a total of 31 sites along the river, including 11, 10, 7, and 3 sites in the Sections 1, 2, 3, 4, respectively. Zooplankton and water samples were collected using the methods of Xiong et al. (2017). At each site, 60 L water from the bottom to water surface was collected and filtered through a 20‐μm mesh net. Zooplankton samples were immediately preserved in 100% alcohol with a volume of 100 ml. Meanwhile, 500 ml water was collected for water chemistry analysis. All samples were stored and transported to the laboratory at 4°C. Geographical locations of each site were recorded using a Garmin Handheld GPS navigator (Garmin Ltd., Kansas, USA).

### 2.2 Environmental variables

For each site, we measured water temperature (T), electric conductivity (EC), pH, total dissolved solid (TDS), and oxidation–reduction potential (ORP) in the field using a multiparameter water quality sonde (MYRON L Company, CA, USA). A portable dissolved oxygen meter (HACH Company, CO, USA) and a Handheld Fluorometer (Turner Designs, CA, USA) were used to record dissolved oxygen (DO) and concentration of Chlorophyll a (Chl_a), respectively. Moreover, we measured the concentration of total nitrogen (TN), nitrate (NO_3_‐N), ammonium (NH_4_‐N), total phosphorous (TP), soluble reactive phosphorous (SRP), and chemical oxygen demand (COD) using the methods described by Xiong et al. (2017). We also measured the concentration of potassium (K), calcium (Ca), sodium (Na), and magnesium (Mg) using inductively coupled plasma optical emission spectrometry (PerkinElmer Inc., MA, USA).

### 2.3 DNA extraction, PCR amplification, and high‐throughput sequencing

The total genomic DNA of all zooplankton communities was extracted using the DNeasy Blood and Tissue Kit (Qiagen N.V., Hilden, Germany). Before DNA extraction, bottles that contained zooplankton samples were shaken to homogenize individuals. The quality and quantity of DNA extracts were measured with a NanoDrop ND‐2000 UV‐Vis spectrophotometer (NanoDrop Tech., DE, USA).

The DNA extracts were used as PCR templates to amplify the V4 region of nuclear small subunit ribosomal DNA with the primer pair of Uni18S/Uni18SR, which was specifically designed for zooplankton communities (Zhan et al., 2013). The primers for each sample were labeled with an addition of a unique eight nucleotide tag at the 5′‐end to allow pooling all samples together. A total of eight replicates were performed for each sample to avoid biased amplification (Xiong et al., 2016; Zhan, Bailey, Heath, & MacIsaac, 2014). PCR amplification was performed in a total volume of 25 μl, and each replicate contained 100 ng of genomic DNA, 1 U of *Taq* polymerase (Takara Holdings Inc., Shiga, Japan), 1 × buffer, 2 mM of MgCl_2_, 0.25 mM of each dNTP, and 0.1 μM of forward and reverse primers, respectively. After denaturation at 95°C for 5 min, 35 cycles of 30 s at 95°C, 30 s at 50°C, 90 s at 72°C were performed, followed by a final elongation step of 10 min at 72°C. Eight replicates of each sample were then pooled together and purified using the Sangon Column PCR Product Purification Kit (Sangon Biotech, Shanghai, China). Finally, the equimolar PCR products of 31 samples were pooled together to ensure equal contribution of all samples. High‐throughput sequencing was conducted using an Illumina Miseq PE 300 platform.

### 2.4 Bioinformatics analysis

Raw sequences were processed using the UPARSE algorithm embedded in USEARCH (Edgar, 2013). Artificial primers and tags were trimmed before subsequent analyses with Python scripts provided by USEARCH. We removed sequences (i) that contained any undetermined nucleotide (N's); (ii) that had Phred quality scores (Q) lower than 20; (iii) that had the maximum expected error threshold lower than 0.75 (Edgar, 2013). Filtered sequences were subsequently trimmed to the same length of 225 bp (Xiong et al., 2017). Operational Taxonomic Units (OTUs) were clustered at a similarity of 97% with dereplicated sequences (Zhan et al., 2014). By searching against the nucleotide database of GenBank online using BLASTn, the taxonomic information of each OTU was obtained. All OTUs and representative sequences were filtered with parameters of *e*‐value <10^−80^, minimum query coverage >80%, and similarity >85% (Zhan et al., 2014). OTUs that were assigned as metazoan zooplankton were kept for further analyses, as the primer pair (Uni18S/Uni18SR) may not well amplify protozoan. As suggested by Xiong et al. (2017) and Sun et al. (2015), the number of sequences was used as the proxy for abundance of each OTU.

### 2.5 Spatial variable analysis

Based on the recorded geographical coordinates, we calculated spatial variables using the Principal Coordinates of Neighbor Matrices (PCNM) analysis (Borcard & Legendre, 2002; Legendre, 2008). This method was conducted *via* eigenvector decomposition which could create a truncated matrix of geographic distances with longitude and latitude of each site. Spatial explanatory variables were selected out when they corresponded to positive eigenvectors and showed positive spatial correlation. Among 20 eigenvectors with positive eigenvalues in our study, 10 were modeled as positive spatial correlation and were used as spatial variables for further analyses. The PCNM analysis was performed using the ‘pcnm’ function in R (R Core Team, 2015).

### 2.6 Statistical analysis

Before statistical analyses, all measured environmental variables, except for pH, were log_10_(*x* + 1) transformed to improve the normality. We then used the analysis of similarity (ANOSIM) and principal component analysis (PCA) to calculate and display the extent of dissimilarity of environmental conditions at all sites.

We used ANOSIM and nonmetric multidimensional scaling (NMDS) with OTU table to compare the variation of zooplankton community among the four sections (Clarke, 1993; Clarke & Gorley, 2001). We also applied analysis of similarity percentages (SIMPER) to select representative OTUs which contributed dominantly to the variation among four sections, and a heat map was produced with ‘*pheatmap*’ in R. All these analyses, including ANOSIM, PCA, NMDS, and SIMPER, were conducted in PRIMER 5.0 (Clarke & Gorley, 2001).

In addition, the redundancy analysis (RDA) was applied to test the significance of the influence of environmental and spatial variables on zooplankton community structure. The RDA was chosen based on a detrended correspondence analysis (DCA) which indicated that the longest gradient length (2.181) was shorter than four, suggesting that the majority of taxa exhibited linear response to variables (Lepš & Šmilauer, 2003). Before RDA, the forward selection was conducted to select variables which were relatively more important among all environmental and spatial factors. DCA and RDA were performed with CANOCO 4.5 package (Lepš & Šmilauer, 2003).

In order to dissect the relative importance of species sorting versus dispersal in affecting the geographical distribution patterns of zooplankton communities, we conducted variation partitioning analysis (Borcard, Legendre, & Drapeau, 1992). The total variation contained four ingredients: the variation explained purely by environmental factors (Env.), the variation explained purely by spatial factors (Spa.), the variation explained jointly by environmental and spatial factors (Env. & Spa.), and the unexplained variation. The total explained variation was calculated through the combination of forward selected environmental and spatial variables, and subsequently, the fractions purely explained by environmental and spatial variables were calculated with partial redundancy analysis (pRDA). During the analysis, Monte Carlo permutation test was conducted to get the significance. The variation partitioning was performed with ‘*varpart* function in R.

Finally, Threshold Indicator Taxa ANalysis (TITAN) was performed in R to get an deeper understanding of changes in taxon distributions along the environmental gradient and to further examine the influence of environmental variables on community structure (Baker & King, 2010). For each taxon, indicator values (IndVal; estimating the association between each taxon with each group) were calculated for all possible change points long the environmental gradient. Permuted IndVal scores were then standardized as z scores and summed for positive and negative values for each change point. Sum(z) peaks highlight the community threshold around which many taxa exhibit strong directional changes in abundance. Before TITAN analysis, taxon abundance was log_10_(*x* + 1) transformed to reduce the influence of high relative abundance taxa. Only taxa with >5 occurrence were included in the analysis. More detailed information on this method could be found in Baker and King (2010).

## 3 RESULTS

### 3.1 Environmental gradient along the river

**Figure 2:**
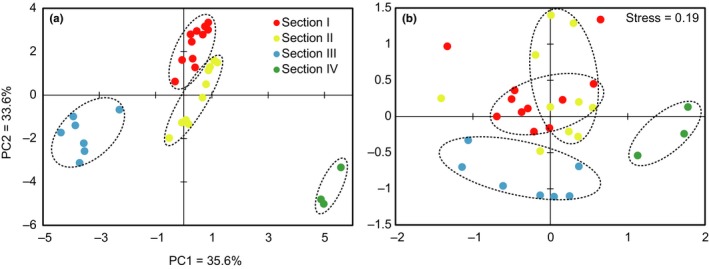
The results of principal component analysis (PCA) of environmental variables at all sites based on the Euclidean distance (a) and the results of nonmetric multidimensional scaling ordination (NMDS) of metazoan zooplankton communities at all sites based on the Bray–Curtis distance (b)

The plot of principal component analysis (PCA) showed that sampling sites were clearly separated into four distinctive clusters (Fig. 2a). Based on this, the whole river could be divided into four sections. Significant changes of environmental variables were observed along the environmental gradient (Table S1). For example, the concentration of total nitrogen (TN), total phosphorous (TP), ammonium (NH_4_‐N), and other nutrient indexes decreased from the Sections 1, 2, 3, but slightly increased in the Section IV. The concentration of dissolved oxygen (DO) was highest (2.76–30.00 mg/L, mean = 16.64 mg/L) in the Section II, while the concentrations of Na, K, Ca, and Mg were highest in the Section IV (Table S1). When the section division was subjected for the confirmation by ANOSIM, the global *r* was 0.706 (*p *=* *.001), and significant difference was detected between each section pair (Table S2).

### 3.2 Community composition and geographical distribution

The high‐throughput sequencing generated a total of 3,809,353 sequences (NCBI SRA No.: PRJNA398157). After OTU clustering and taxonomic annotation, 237 OTUs were identified as metazoan zooplankton species. In addition, the rarefaction curve for each site suggests current sequencing depth is sufficient to capture most of the biodiversity (Fig. S1).

At the phylum level, 59.5% and 37.9% of sequences belonged to Arthropoda and Rotifera, while the relative abundance of each phylum differed in the four sections (Fig. 1). The Section IV harbored a higher abundance of Rotifera than the other three sections, while both Sections I and II contained a higher abundance of Arthropoda. At the genus level, more than 92% of sequences could be assigned to 15 genera with the percentage greater than 1% (Fig. S2a). Dominant genera included *Thermocyclops* (17.5%), *Sinantherina* (17.3%), *Brachionus* (13.6%), *Daphnia* (6.4%), *Pseudodiaptomus* (6.4%), and *Eucyclops* (6.2%). The discrepancy of community composition was also observed at the genus level. For example, we found the highest relative abundance of *Brachionus* (33.8%), *Sinantherina* (28.5%), and *Pseudodiaptomus* (26.2%) in the Section IV (Fig. S2b).

The significant dissimilarity of community structure in the four sections was also detected by NMDS and ANOSIM. The NMDS results showed that both Sections III and IV were clearly separated from the other two groups (Fig. 2b). Further tests based on ANOSIM confirmed such a clustering pattern (global *r *=* *.338; *p *=* *.001), and the intergroup dissimilarity was larger than the intragroup dissimilarity. The community structure was not significantly different between the Sections I and II (*r *=* *0.085, *p *=* *.092), but significant difference was detected between all the other section pairs (*p *<* *.05 for all comparisons). Based on the SIMPER analysis, we detected the dominant OTUs which contributed to the intergroup difference (Fig. S3). For example, the highest relative abundances of OTU2 (Rotifera: *Brachionus*) and OTU3 (Rotifera: *Sinantherina*) were found in the Section IV, while the highest relative abundance of OTU1 (Arthropoda: *Thermocyclops*) was found in the Section III.

### 3.3 Variables responsible for spatial variation in community structure

**Figure 3:**
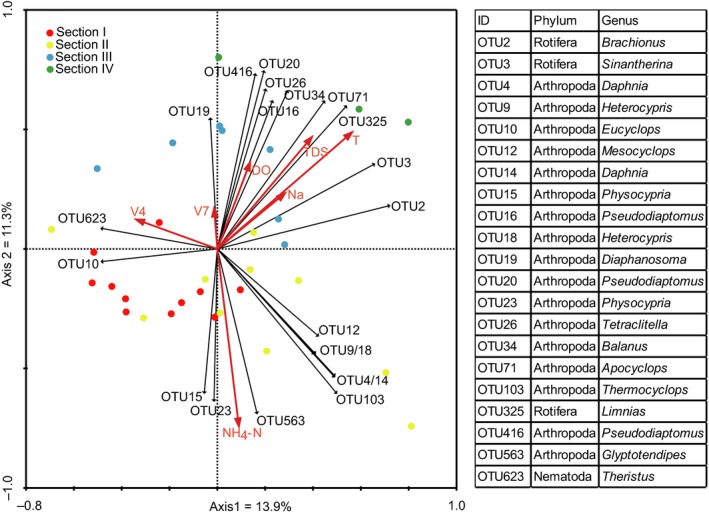
The ordination plot of redundancy analysis (RDA) of metazoan zooplankton communities at all sites. Species weakly associated with the first two axes (with fitness <20%) and with the occurrence at <30% of sites were omitted for clarity. Black and red arrows represent Operational Taxonomic Units (OTUs) and measured variables, respectively. The right table shows the phylum and genus of each OTU

Through forward selection, five variables, including NH_4_‐N, Na, TDS, DO, and T, were selected out of all 17 measured environmental variables, while V7 and V4 were selected out of the 20 spatial variables to explain zooplankton community structure. Values of environmental variables revealed apparent changes of each factor along the river (Fig. S4). Using RDA, we combined these selected variables to test the relationship between explanatory variables and community distribution patterns. Our results showed that NH_4_‐N was the most important environmental factor that influenced geographical distribution of zooplankton, followed by Na, TDS, DO, and T (Fig. 3). The community structure varied along the gradient of NH_4_‐N, with communities at the Sections I and II surviving the high concentration of NH_4_‐N, while species at the Sections III and IV living at the low concentration of NH_4_‐N (Fig. 3). For spatial variables, V7 was the most important one responsible for the varied community structure, followed by V4 (Fig. 3). In general, V7 modeled the spatial distribution of metazoan communities along the river. V4 modeled a finer spatial scale in Sections I and II, but not related to Sections III and IV.

### 3.4 Determinative roles of species sorting versus dispersal

**Figure 4:**
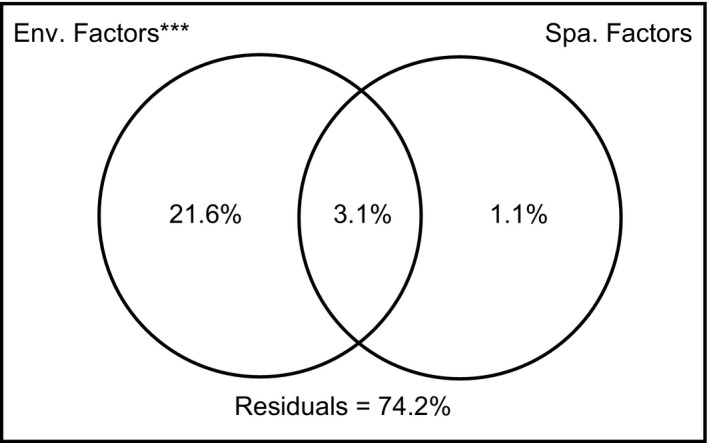
Results of variation partitioning. Environmental variables and spatial variables explained 21.6% and 1.1% of the total variation, respectively

As both environmental and spatial variables could affect community structure, we used variation partitioning to analyze the relative importance of these two groups of explanatory variables. Our results revealed that environmental variables alone explained 21.6% of all the variation, while a much lower proportion (1.1%) could be explained by spatial variables (Fig. 4).

### 3.5 Responses of taxon and community assemblages to nutrient

**Figure 5:**
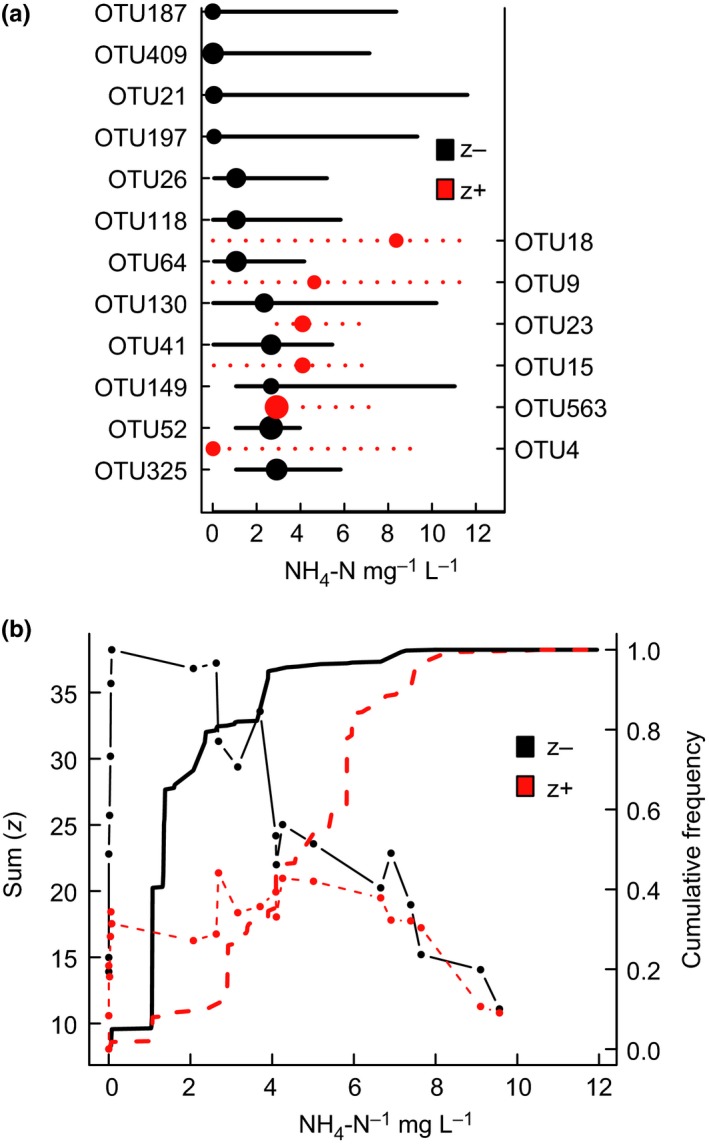
Threshold Indicator Taxa ANalysis (TITAN) of zooplankton community response to the gradient of ammonium (NH4‐N). (a) Pure (≥0.95) indicator taxa are plotted in increasing order with respect to their observed environmental change point. Black symbols correspond to negative (z−) indicator taxa, while red corresponds to positive (z+) indicator taxa. Symbols are scaled in proportion to z scores. Horizontal lines overlapping each symbol represent 5th and 95th percentiles among 500 bootstrap replicates. (b) TITAN sum(z−) and sum(z+) values corresponding to all candidate change points along the environmental gradient. Black and red vertical lines represent the cumulative frequency distribution of change points among 500 bootstrap replicates for sum(z−) and sum(z+), respectively

We used TITAN to further analyze the response of zooplankton taxon and community to the most important environmental variable—NH_4_‐N. At the taxon level, TITAN identified 12 OTUs as negative (z−) indicator taxa that decreased with NH_4_‐N concentration between 0.002 and 3.0 mg/L, and only six OTUs were identified as positive (z+) indicators that increased with NH_4_‐N gradient between 0.002 and 8.0 mg/L (Fig. 5a). Most individual taxa change points overlapped at the 0.002–6.0 mg/L range, suggesting the existence of an ecological community threshold (Table S3). At the community level, the results of TITAN revealed a sum(z−) change point of 1.074 mg/L and a distinct sum(z+) change point at 2.922 mg/L (Fig. 5b; Table 1).

**Table 1:** TITAN community‐level thresholds estimated from zooplankton taxa responses to gradient of ammonium

Gradient	Method	Change points			
		Obs.	5%	50%	95%
NH_4_‐N (mg/l)	Sum(z−)	1.074	0.989	1.353	4.105
	Sum(z+)	2.922	1.074	4.633	7.518

TITAN observed change points (obs.) correspond to the value of the nutrient gradient resulting in the largest of sum of indicator value (IndVal) z scores among all negative (z−) and positive (z+) taxa, respectively. Quantiles (5%, 50%, 95%) correspond to the change points from 1000 bootstrap replicates.

## 4 DISCUSSION

### 4.1 The importance of species sorting over dispersal in highly polluted river ecosystems

In freshwater ecosystems, numerous studies have tried to disentangle the relative importance of species sorting versus dispersal to determine meta‐community assemblages (e.g., Mykrä, Heino, & Muotka, 2007; Xiong et al., 2017). So far, it has been well‐known that the roles of these two processes are scale‐dependent (Heino, Melo, Siqueira, et al., 2015). At large spatial scales such as across basins, the dispersal limitation precludes species to occur at suitable habitats effectively; while at fine geographical scales such as within a stream or river, environmental conditions are expected to be homogenized by water flows, which makes dispersal overwhelmingly dominant in structuring communities (Heino, Melo, Siqueira, et al., 2015). However, our study in NCR revealed the stronger influence of species sorting (21.6%) than dispersal (1.1%) in structuring metazoan communities’ composition and distribution. Our study provides direct evidence and support for the “fine‐scale species sorting hypothesis” proposed by Xiong et al. (2017). Both Xiong et al. (2017)'s and our studies revealed dispersal to be less important than species sorting in highly polluted running water ecosystems, which is in contrast to the conclusions in previous studies of relatively less polluted water ecosystems (Heino & Grönroos, 2013). The major feature contrasting these two studies differ from others is the existence of a sufficient environmental gradient created by local pollution. Both Chaobai River in Xiong et al. (2017)'s study and NCR are located in the Haihe River Basin, which is the most severely polluted river basin in China (see references in Xiong et al., 2017). For NCR, the gradient of environmental variables is mainly due to the different pollution sources, with relatively more point pollution from industrial and domestic wastewater discharge in the upper and lower streams, while relatively more agricultural diffuse pollution from farming lands in the middle stream. The selection pressure exerted by pollution pushed species towards more preferable habitats in spite of potential dispersal ability (Xiong et al., 2017), which leads to significantly different assemblies of metazoan community in different sections (Fig. 2b). Thus, the environmental heterogeneity created by environmental gradient is the fundamental cause for the dominance of species sorting over dispersal at NCR.

Although both studies support the fine‐scale species sorting hypothesis, the comparison of these two studies revealed that different environmental factors could influence zooplankton communities in different rivers with varied pollution levels. In Xiong et al.'s study, the concentration of nutrients (including nitrogen, phosphorous and COD) was relatively lower in Chaobai River, especially in the mountain area where the river is originated (see more details in Xiong et al., 2017). However, NCR in this study mainly served as the wastewater‐receiving river of Beijing and Tianjin, and the level of pollution was relatively more severe and complex (Heeb et al., 2012). The complexity of pollution might contribute to the low percentage of explained variation as many pollutants are unknown and difficult to measure. It is possible that some undetected variables can also influence the community structure of metazoan communities. However, it is impossible to capture all biotic and abiotic variables in rivers with complex chemical pollution. Our study also suggests that environmental factors responsible for biological community structure in different rivers with varied levels of pollution should be identified when restoration programs are planned and implemented.

### 4.2 The geographical distribution of metazoan zooplankton communities

The relative abundance of Arthropoda (mostly crustaceans) decreased from upstream to downstream, coupled with the increasing abundance of Rotifera. The reverse relationship of these two groups has been commonly found in the field (Fussmann, 1996; Wang, Xie, & Geng, 2010). One of the most important reasons is the competition of rotifers and crustaceans for food resources. With larger body size and stronger mobility, crustaceans could outcompete rotifers to get more resources (MacIsaac & Gilbert, 1989, 1991), and rotifers may be constrained to less preferable habitats. For example, in our study, the relative abundance of rotifers increased from Sections I and II to Section III where the concentration of Chl_a was lowest. The other reason might be due to predation, as some crustaceans could prey on small sized rotifers, leading to the higher abundance of crustaceans while the lower abundance of rotifers (Laxson, McPhedran, Makarewicz, Telesh, & MacIsaac, 2003; Meyer, Hampton, Ozersky, Rusanovskaya, & Woo, 2017). Thus, species interactions need to be taken into consideration as one crucial factor responsible for changes of community structure in future studies.

Among the four segments, metazoan communities were not significantly different between Sections I and II, although the corresponding environmental factors significantly varied. This is likely due to the short gradient of the selected dominant factors (Fig. S4). This finding suggests that the relative contribution of each variable to community structure is unequal, and minor difference of environmental factors was easy to be masked by the integration of all variables. Thus, forward selection was crucial to provide a useful method to select dominant factors (Blanchet, Legendre, & Borcard, 2008). This method could facilitate the understanding of potential influences of environmental variables on community structure.

### 4.3 Contribution of environmental variables to meta‐community composition and structure

Environmental factors have long been recognized as important drivers influencing freshwater ecosystems, especially the concentration of nitrogen and phosphorus (Elser et al., 2007; Smith & Schindler, 2009). The high concentration of nutrients could promote the production of primary producers, which in turn would facilitate the reproduction and growth of zooplankton and other predators that feed on them (Fermani et al., 2013). Similarly, our study found that the concentration of nutrients contributed greatly to zooplankton community structures, especially the ammonium (NH_4_‐N; Fig. 3). As revealed by the results of TITAN, we also found a community‐level nutrient threshold of zooplankton responses to the concentration of NH_4_‐N (Fig. 5b), which split community into either positive [sum(z+)] or negative [sum(z−)] groups. This result revealed the existence of nonlinear responses of zooplankton community along the environmental gradient. Being a highly polluted river, the excess loads of nutrients in NCR mainly result from treated and untreated wastewater (Heeb et al., 2012). As a result, geographical distributions of other nutrients, including TN, NO_3_‐N, TP, and SRP, were consistent with NH_4_‐N. Consequently, pollution‐mediated nutrient enrichment represents the important driver of community structure of zooplankton.

We also investigated the taxon‐level responses to the concentration gradient of NH_4_‐N. We found that different species responded differently to the concentration of NH_4_‐N. As revealed by TITAN, six out of 18 indicator taxa responded positively to the increasing concentration of NH_4_‐N (Fig. 5a). This pattern is largely attributed to toxicity effects of NH_4_‐N and species’ tolerance. Ammonium is actually composed of unionized (NH_3_‐N) and ionized (NH_4_‐N) ammonium, two types which show mutual transformation due to changes of pH and temperature. The form of NH_4_‐N is biologically safe, while NH_3_‐N can pass through the biological surface of organisms and permeate into their bodies, resulting in ammonium intoxication (Cheng et al., 2015; Yang et al., 2017). However, different species have different tolerance to ammonium. The ecotoxicology assay showed that one species of *Physocypria* (*P. kraepelini*) could survive for a long term when the concentration of ammonium was less than 58.38 mg/L, and the LC_50_ value for 96 h exposure was 583.82 mg/L (Yu, Chen, Li, Chen, & Chen, 2009). Such a high tolerance to ammonium may help us understand the distribution of *Physocypria* (OTU15, OTU23) at the high concentration of ammonium (Fig. 3) and their positive responses to the concentration of NH_4_‐N (Fig. 5a). However, 12 out of 18 indicator taxa showed signally decreasing trend of abundance along the environmental gradient and the thresholds were much lower than the laboratory experiments. This finding highlighted the severity of pollution in the North Canal River. Consequently, species occurring in certain areas suggests their adaptive responses to local environment. Shifts of zooplankton community composition and structure could be effective bio‐indicators of environmental changes (Fermani et al., 2013; Xiong et al., 2017).

## 5 CONCLUSIONS

This study provides direct evidence to support the newly proposed “fine‐scale species sorting hypothesis”, with the existence of a significant environmental gradient formed by different environmental variables linked with varied human activities along the river and significant variation of meta‐community structures in different sections. Multiple analyses suggest that species sorting, rather than dispersal, was relatively more important in structuring community composition and geographical distribution. We propose the common presence of species sorting at fine geographical scales when significant environmental gradients exist. In addition, our study suggests that the geographical distribution of meta‐community is driven by different levels of selection pressure exerted by anthropogenic activity‐mediated pollution. Thus, further investigations of meta‐community dynamics should take into consideration of not only possible mechanisms underlying community composition and distribution, but also the impacts and treatments of water pollution. Finally, species interactions and tolerance mechanisms of species to certain pollutants also deserve more efforts to better understand the biological and ecological processes relating to the meta‐community dynamics.

## CONFLICT OF INTEREST

None declared.

## AUTHOR CONTRIBUTIONS

AZ conceived the study. AZ and YY designed the experiment. YY, PN, YG and WX conducted the experiments. YY, PN, YG, WX and YZ analyzed the data. YY, PN, YG, WX and AZ wrote the manuscript. All authors reviewed and commented on the manuscript.

## DATA AVAILABILITY

The high‐throughput sequencing data was deposited into NCBI GenBank (SRA No.: PRJNA 398157).
