# Supplementary material for: Geographical distribution of zooplankton biodiversity in highly polluted running water ecosystems: Validation of fine‐scale species sorting hypothesis
Source: Ecol Evol. 2018 Apr 17;8(10):4830–40. doi: 10.1002/ece3.4037 (PMC5980572; doi:10.1002/ece3.4037)
Supplement: Supplementary file 1 [file ECE3-8-4830-s001.doc]

# Supplementary materials


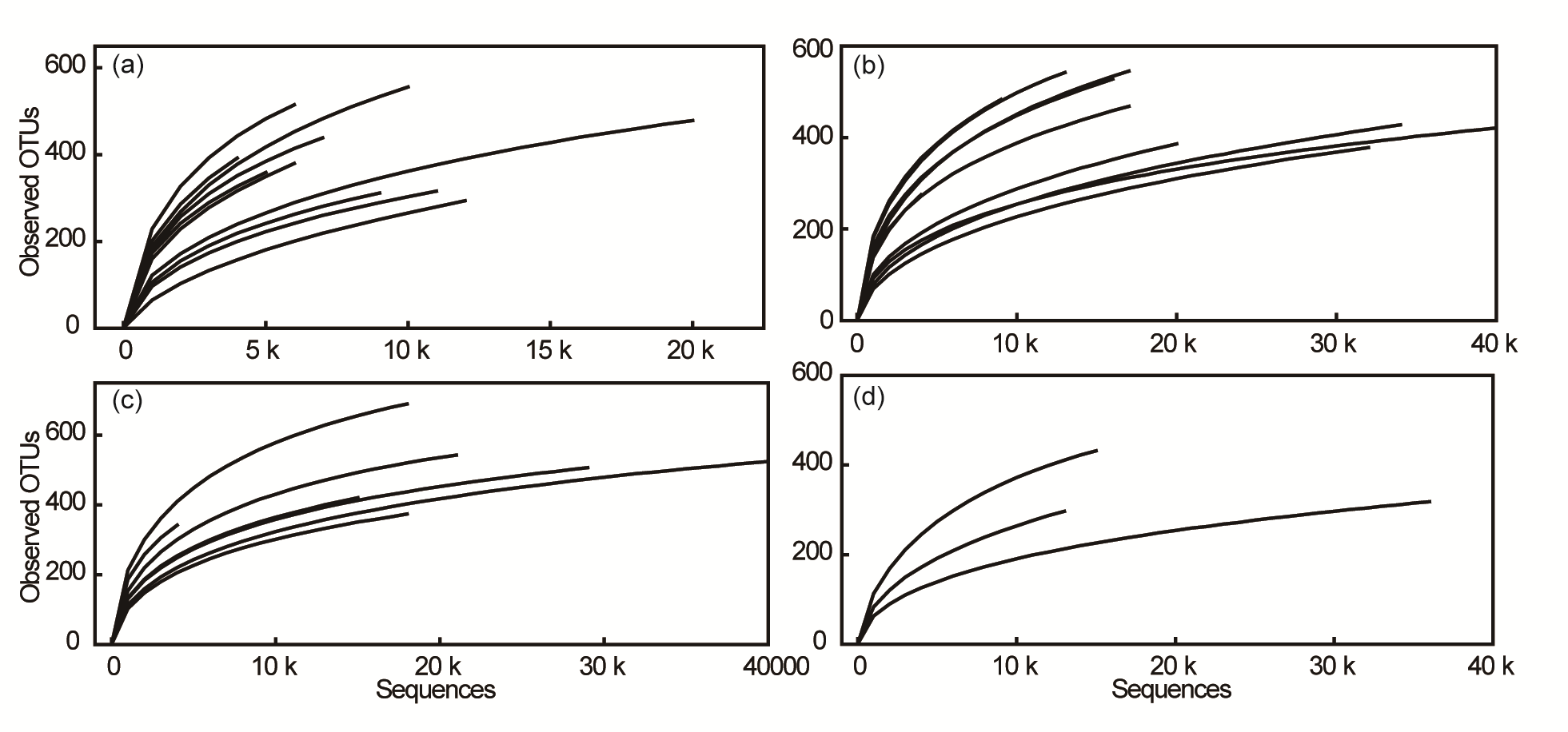


Figure S1. Rarefaction curves of all sites in the four sections: (a) Section I, (b) Section II, (c) Section III and (d) Section IV.

**
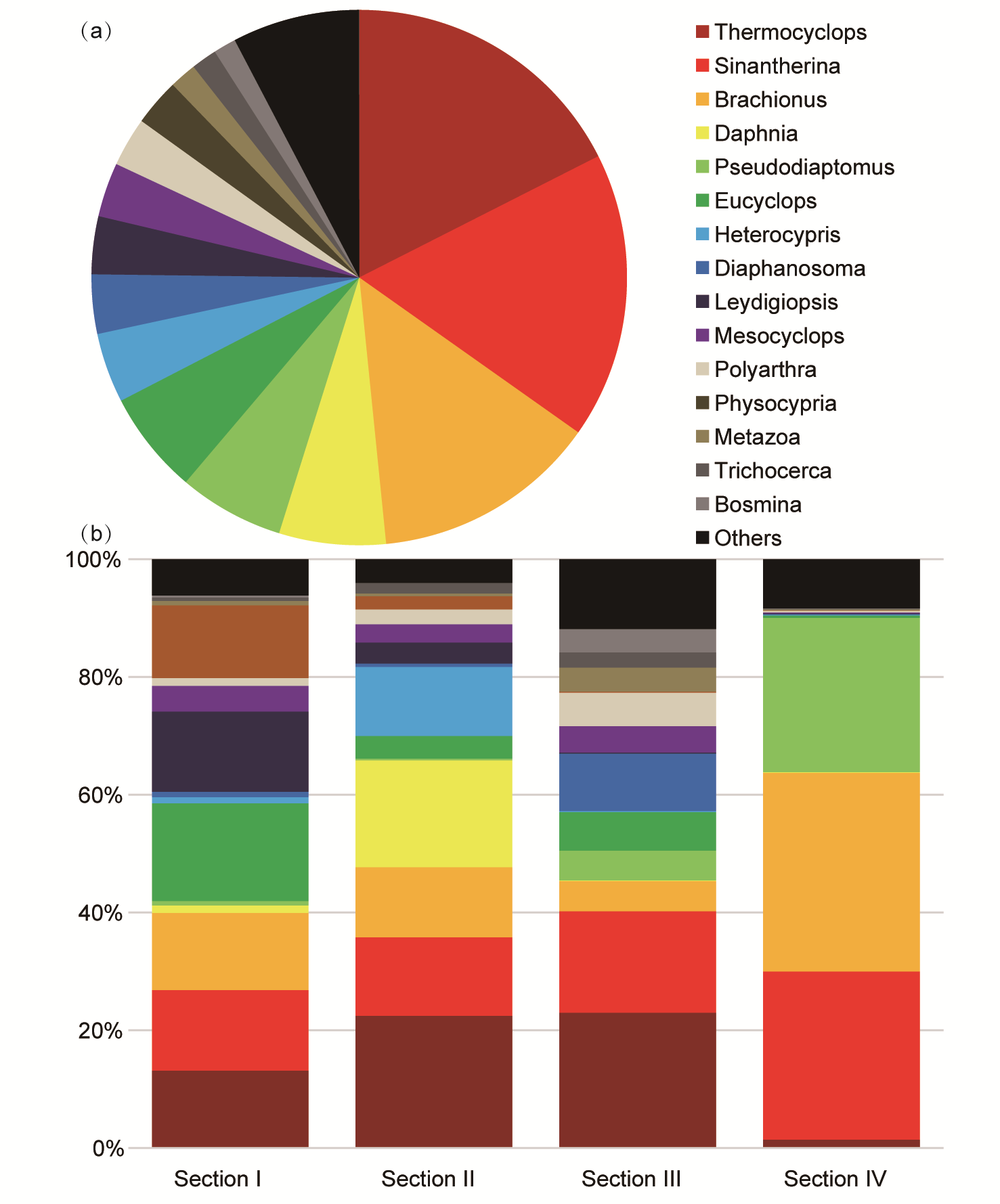
**

Figure S2. The genus-level composition of metazoan zooplankton communities at all sites (a) and in four sections (b).

**
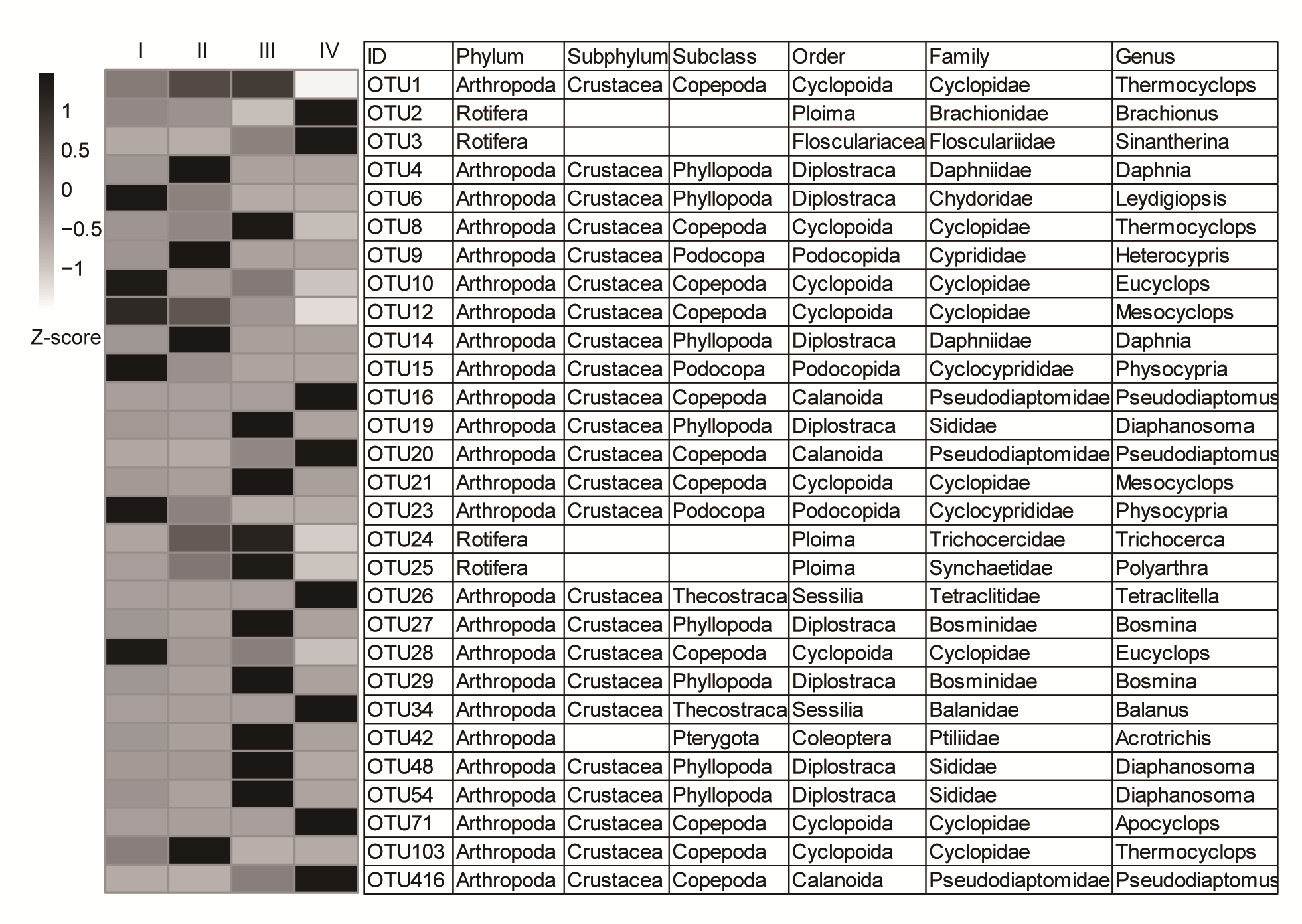
**

Figure S3. Heatmap of relative abundances of dominant OTUs that contributed to the 50% of community differences among the four sections. These OTUs were selected using Similarity Percentages (SIMPER) using PRIMER 5.0. Table on the right showed the taxonomic information of each OTU. OTU = Operational Taxonomic Units.

**
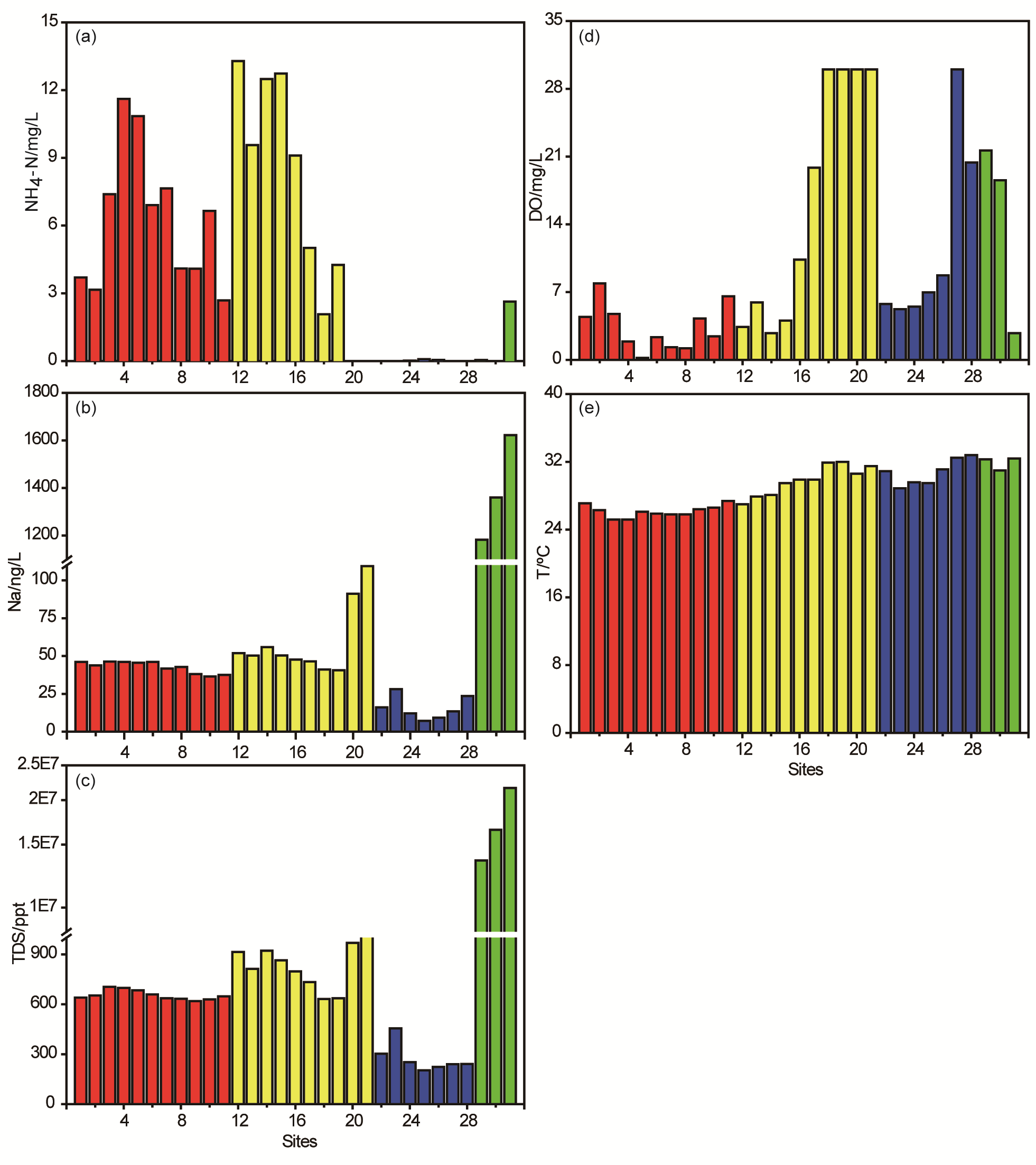
**

Figure S4. Histograms of selected environmental factors at all sites. There were five variables in total, including (a) ammonium nitrogen (NH_4_-N), (b) sodium (Na), (c) total dissolved solid (TDS), (d) dissolved oxygen (DO) and (e) temperature (T). The red, yellow, blue and green columns represent the Sections I- IV, respectively.

Table S1. The minimum, maximum, and mean values of environmental factors at all sites. TN = Total Nitrogen; NH_4_-N = Ammonium Nitrogen; NO_3_-N = Nitrate Nitrogen; TP = Total Phosphorus; SRP = Soluble Reactive Phosphorus; COD = Chemical Oxygen Demand; K = Potassium; Ca = Calcium; Na = Sodium; Mg = Magnesium; Chl_a = Chlorophyll a; EC = Electric Conductivity; TDS = Total Dissolved Solid; ORP = Oxidation-Reduction Potential; DO = Dissolved Oxygen; T = Temperature.

|  | Section I | | | Section II | | | Section III | | | Section IV | | |
| --- | --- | --- | --- | --- | --- | --- | --- | --- | --- | --- | --- | --- |
|  | Min. | Max. | Mean | Min. | Max. | Mean | Min. | Max. | Mean | Min. | Max. | Mean |
| TN/mg/L | 14.89 | 60.06 | 40.37 | 14.89 | 32.56 | 23.89 | 4.14 | 19.26 | 10.49 | 10.15 | 20.90 | 14.95 |
| NH_4_-N/mg/L | 2.69 | 11.61 | 6.25 | 0.002 | 13.29 | 6.85 | 0.002 | 0.08 | 0.02 | 0.002 | 2.63 | 0.89 |
| NO_3_-N/mg/L | 5.29 | 40.99 | 13.83 | 5.29 | 25.39 | 10.45 | 1.66 | 4.01 | 2.97 | 1.87 | 2.94 | 2.44 |
| TP/mg/L | 2.60 | 10.53 | 5.09 | 3.23 | 4.83 | 3.95 | 0.31 | 1.26 | 0.75 | 1.69 | 1.74 | 1.73 |
| SRP/mg/L | 1.81 | 6.73 | 4.13 | 1.69 | 4.09 | 3.02 | 0.00 | 0.19 | 0.03 | 0.97 | 1.27 | 1.07 |
| COD/mg/L | 33 | 335 | 94 | 25 | 118 | 61 | 0 | 124 | 70 | 34 | 128 | 88 |
| K/μg/L | 7.44 | 10.76 | 8.99 | 5.19 | 10.07 | 8.34 | 1.48 | 2.66 | 1.88 | 53.40 | 76.97 | 64.39 |
| Ca/μg/L | 27.21 | 30.70 | 28.86 | 23.25 | 40.71 | 34.64 | 10.17 | 21.58 | 16.06 | 67.48 | 95.55 | 79.67 |
| Na/μg/L | 36.49 | 46.28 | 42.78 | 40.60 | 109.46 | 58.47 | 7.21 | 28.11 | 15.71 | 1182.87 | 1622.17 | 1388.39 |
| Mg/μg/L | 19.20 | 22.90 | 20.81 | 23.86 | 38.44 | 28.60 | 9.76 | 17.10 | 12.70 | 210.67 | 252.76 | 231.31 |
| Chl_a/μg/L | 7.4 | 78.5 | 22.1 | 18.2 | 121.5 | 56.9 | 4.8 | 19.6 | 12.3 | 4.3 | 112.8 | 73.2 |
| EC/μs/cm | 915.70 | 1034.00 | 966.47 | 944.30 | 1601.00 | 1230.22 | 319.00 | 688.70 | 423.60 | 15710.00 | 23410.00 | 19243.33 |
| TDS/μg/L | 618.4 | 704.8 | 655.0 | 632.3 | 1107.0 | 838.9 | 203.9 | 455.4 | 274.5 | 13.6(mg/L) | 21.6(mg/L) | 17.2(mg/L) |
| ORP/mv | 36.00 | 224.00 | 178.55 | 50.00 | 172.00 | 113.10 | 44.00 | 128.00 | 89.71 | 58.00 | 70.00 | 63.33 |
| DO/mg/L | 0.21 | 7.91 | 3.40 | 2.76 | 30.00 | 16.64 | 5.24 | 30.00 | 11.80 | 2.77 | 21.63 | 14.32 |
| T/ºC | 25.2 | 27.4 | 26.1 | 27.0 | 32.0 | 29.8 | 28.9 | 32.8 | 30.7 | 31.0 | 32.4 | 31.9 |
| pH | 7.6 | 8.5 | 7.9 | 7.9 | 9.5 | 8.6 | 7.4 | 9.7 | 8.5 | 8.1 | 9.1 | 8.7 |

Table S2. Results of analysis of similarity (ANOSIM) of environmental factors and metazoan zooplankton communities in the four sections.

| Environmental factors | | | | Communities | | | |
| --- | --- | --- | --- | --- | --- | --- | --- |
| Group 1 | Group 2 | R | *P* value | Group 1 | Group 2 | R | *P* value |
| Section I | Section II | 0.31 | 0.005** | Section I | Section II | 0.085 | 0.092 |
|  | Section III | 0.931 | 0.001*** |  | Section III | 0.324 | 0.004** |
|  | Section IV | 1.0 | 0.003** |  | Section IV | 0.77 | 0.003** |
| Section II | Section III | 0.806 | 0.001*** | Section II | Section III | 0.324 | 0.005** |
|  | Section IV | 1.0 | 0.003** |  | Section IV | 0.621 | 0.003** |
| Section III | Section IV | 1.0 | 0.008** | Section III | Section IV | 0.845 | 0.008** |
| Global test | | 0.706 | 0.001*** |  | Global | 0.338 | 0.001*** |

Table S3. Declining (z-) and increasing (z+) taxa results from Threshold Indicator Taxa ANalysis (TITAN) of zooplankton community in response to NH_4_-N (mg/L) in the North Canal River. Only taxa that met significant criteria for p (≤ 0.05), purity (≥ 0.95), and reliability (≥ 0.95) are included in this table. Indicator scores (IndVal and z), and frequency of occurrence (Freq.).

| ID | Taxonomy | +/- | Change point (NH4-N, mg/L) | | | | z | IndVal | p | Purity | Reliability | Freq. |
| --- | --- | --- | --- | --- | --- | --- | --- | --- | --- | --- | --- | --- |
|  |  |  | Obs. | 5% | 50% | 95% |  |  |  |  |  |  |
| OTU187 | Metazoa | z- | 0.002 | 0.002 | 2.352 | 8.374 | 3.17 | 62.76 | 0.004 | 0.994 | 0.976 | 14 |
| OTU409 | Rotifera / Keratella | z- | 0.011 | 0.002 | 0.02 | 7.151 | 4.47 | 68.01 | 0.008 | 0.992 | 0.956 | 12 |
| OTU21 | Arthropoda / Mesocyclops | z- | 0.048 | 0.031 | 1.074 | 11.615 | 3.54 | 65.50 | 0.004 | 0.996 | 0.978 | 30 |
| OTU197 | Metazoa | z- | 0.064 | 0.031 | 0.064 | 9.333 | 2.91 | 66.96 | 0.004 | 0.992 | 0.956 | 29 |
| OTU26 | Arthropoda / Tetraclitella | z- | 1.074 | 0.064 | 2.659 | 5.210 | 4.08 | 75.09 | 0.004 | 0.996 | 0.982 | 20 |
| OTU118 | Arthropoda / Neodiaptomus | z- | 1.074 | 0.011 | 1.353 | 5.834 | 3.87 | 67.39 | 0.008 | 0.990 | 0.950 | 16 |
| OTU64 | Arthropoda / Eurytemora | z- | 1.074 | 0.059 | 1.074 | 4.178 | 4.38 | 73.18 | 0.004 | 1.000 | 0.970 | 15 |
| OTU130 | Rotifera / Monostyla | z- | 2.352 | 0.020 | 2.352 | 10.205 | 3.88 | 69.89 | 0.004 | 1.000 | 0.994 | 23 |
| OTU41 | Rotifera / Keratella | z- | 2.659 | 0.031 | 2.659 | 5.456 | 4.23 | 67.04 | 0.004 | 0.998 | 0.998 | 29 |
| OTU149 | Rotifera / Asplanchnopus | z- | 2.659 | 1.063 | 3.665 | 11.038 | 3.14 | 64.32 | 0.008 | 0.994 | 0.986 | 26 |
| OTU52 | Arthropoda / Sinocalanus | z- | 2.659 | 1.046 | 2.659 | 3.980 | 5.05 | 73.49 | 0.004 | 0.986 | 0.976 | 17 |
| OTU325 | Rotifera / Limnias | z- | 2.922 | 1.063 | 3.388 | 5.834 | 4.54 | 57.79 | 0.004 | 1.000 | 0.964 | 12 |
| OTU18 | Arthropoda / Heterocypris | z+ | 8.374 | 0.011 | 5.834 | 11.615 | 3.33 | 65.63 | 0.008 | 0.998 | 1.000 | 31 |
| OTU9 | Arthropoda / Heterocypris | z+ | 4.633 | 0.011 | 5.834 | 11.618 | 3.18 | 59.83 | 0.004 | 0.996 | 0.988 | 31 |
| OTU23 | Arthropoda / Physocypria | z+ | 4.096 | 2.922 | 4.096 | 7.292 | 4.04 | 72.01 | 0.004 | 0.996 | 0.998 | 31 |
| OTU15 | Arthropoda / Physocypria | z+ | 4.096 | 0.053 | 4.096 | 7.163 | 3.76 | 66.62 | 0.004 | 0.996 | 0.996 | 31 |
| OTU563 | Arthropoda / Glyptotendipes | z+ | 2.922 | 2.615 | 4.556 | 7.518 | 6.29 | 64.71 | 0.004 | 1.000 | 0.998 | 11 |
| OTU4 | Arthropoda / Daphnia | z+ | 0.011 | 0.011 | 1.068 | 9.506 | 3.47 | 68.66 | 0.004 | 0.994 | 0.978 | 31 |
